# Constructing a robust protein-protein interaction network by integrating multiple public databases

**DOI:** 10.1186/1471-2105-12-S10-S7

**Published:** 2011-10-18

**Authors:** Venkata-Swamy Martha, Zhichao Liu, Li Guo, Zhenqiang Su, Yanbin Ye, Hong Fang, Don Ding, Weida Tong, Xiaowei Xu

**Affiliations:** 1 Department of Information Science, University of Arkansas at Little Rock, 2801 S. University Ave., Little Rock, AR 72204-1099, USA; 2 Center for Bioinformatics, Division of Systems Biology, National Center for Toxicological Research, US Food and Drug Administration, 3900 NCTR Road, Jefferson, AR 72079, USA; 3 State Key Laboratory of Multiphase Complex Systems, Institute of Process Engineering, Chinese Academy of Sciences, Beijing, 100190, P.R. China; 4 ICF International at FDA's National Center for Toxicological Research, 3900 NCTR Rd, Jefferson, AR 72079, USA

## Abstract

**Background:**

Protein-protein interactions (PPIs) are a critical component for many underlying biological processes. A PPI network can provide insight into the mechanisms of these processes, as well as the relationships among different proteins and toxicants that are potentially involved in the processes. There are many PPI databases publicly available, each with a specific focus. The challenge is how to effectively combine their contents to generate a robust and biologically relevant PPI network.

**Methods:**

In this study, seven public PPI databases, BioGRID, DIP, HPRD, IntAct, MINT, REACTOME, and SPIKE, were used to explore a powerful approach to combine multiple PPI databases for an integrated PPI network. We developed a novel method called *k*-votes to create seven different integrated networks by using values of *k* ranging from 1-7. Functional modules were mined by using SCAN, a Structural Clustering Algorithm for Networks. Overall module qualities were evaluated for each integrated network using the following statistical and biological measures: (1) modularity, (2) similarity-based modularity, (3) clustering score, and (4) enrichment.

**Results:**

Each integrated human PPI network was constructed based on the number of votes (*k*) for a particular interaction from the committee of the original seven PPI databases. The performance of functional modules obtained by SCAN from each integrated network was evaluated. The optimal value for *k* was determined by the functional module analysis. Our results demonstrate that the *k*-votes method outperforms the traditional union approach in terms of both statistical significance and biological meaning. The best network is achieved at *k*=2, which is composed of interactions that are confirmed in at least two PPI databases. In contrast, the traditional union approach yields an integrated network that consists of all interactions of seven PPI databases, which might be subject to high false positives.

**Conclusions:**

We determined that the k-votes method for constructing a robust PPI network by integrating multiple public databases outperforms previously reported approaches and that a value of k=2 provides the best results. The developed strategies for combining databases show promise in the advancement of network construction and modeling.

## Background

Protein-protein interaction (PPI) is a critical component of almost every biological process related to physiological conditions, and can be analyzed in a PPI network to discover underlying mechanisms of toxicity and disease at the integrated system level [1]. A PPI network reflects the mode of action caused by interruptions of the protein and related targets. Crucial PPIs are proven to participate in disease-associated signaling pathways, which can offer insight for novel target identification and drug discovery. With the development of high-throughput molecular technology such as gene expression microarrays and *in vitro* assay screening platforms, analyzing PPIs has become a common strategy to interpret the findings.

For example, many current studies focus on how to mine disease-related genes/proteins to provide a better understanding of the mechanisms of diseases by using PPI databases; the hypothesis is that genes related to the same disease tend to encode proteins that interact with each other [2]. Therefore, PPI data are crucial for new disease biomarker discovery, disease-disease relationship searching, and common biological function detection. However, most research focuses on constructing and evaluating PPI networks based on a single source of PPI data or by using simple unions of multiple PPI databases [3,4]. Although many public PPI databases provide rich information, each database is developed with a specific focus and emphasis, and no single existing database is comprehensive. Therefore, developing methods to integrate PPI databases and construct a robust and biologically relevant PPI network is of great importance. The question is how to combine multiple PPI databases so that the best integrated PPI network can be established.

In this study, seven PPI databases (BioGRID, DIP, HPRD, IntAct, MINT, REACTOME, and SPIKE) were used as case studies to explore a novel approach to effectively combine multiple databases into an integrated PPI network. A structural clustering algorithm for networks (SCAN), was employed to evaluate seven integrated networks resulting from different values for *k*[5]. Statistical and biological measures including modularity, similarity-based modularity, clustering score and enrichment were used to assess the integrated networks [2]. The developed strategies for combining multiple databases show promise for future application in network construction and modeling.

## Methods

### Database construction

For this study, seven PPI databases were downloaded from public domain sources. BioGRID is a publication search-driven database which covers the raw protein data and genetic interactions from major model species such as *Saccharomyces cerevisiae*, *Caenorhabditis elegans*, *Drosophila melanogaster*, and *Homo sapiens*[*6*]. The DIP^TM^ database catalogs experimentally determined interactions between proteins with automatic computational corrections as well as manual reviews performed by experts [7]. HPRD includes around 40,000 PPIs detected through experiments , covering over 30,000 human protein entities [8]. IntAct is a molecular interaction database, either extracted from literature or directly deposited by expert curators following a comprehensive annotation [9]. MINT focuses on experimentally verified PPIs in all species by data-mining scientific literature [10]. REACTOME is an open-source, manually curated, and peer-reviewed pathway database, which provides insight into gene/protein interactions from pathway perspectives and species comparisons [11]. SPIKE is a database of thoroughly curated human signaling pathways [12].

The total number of human proteins, their interactions, and the website page for each database is listed in Table 1. In this study, only *Homo sapiens* proteins have been included in the network construction. The disparate protein IDs in different databases have been consolidated and unified using the Entrez ID.

**Table 1:** Information for the seven public PPI databases

Databases	Number of proteins	Number of interactions	Websites
BioGRID	8204	33625	http://thebiogrid.org
DIP	1137	1509	http://dip.doe-mbi.ucla.edu/dip/Main.cgi
HPRD	9553	38802	http://www.hprd.org
IntAct	7495	30965	http://www.ebi.ac.uk/intact/main.xhtml
MINT	5230	15353	http://mint.bio.uniroma2.it/mint/Welcome.do
REATOME	3599	74490	http://www.reactome.org
SPIKE	6927	23224	http://www.cs.tau.ac.il/~spike/

Given a set of PPI databases, each can be represented by a network consisting of a set of vertices that are connected to each other by a set of edges. In this model, each vertex is a protein; and the interaction between a pair of proteins is an edge in the network. We constructed seven interaction databases in this study; however, our method may be reproduced for any number of databases. In the following, we assume there are *n* networks to be integrated. Our goal is to find an optimal strategy to integrate them for the most robust and biologically significant PPI network. Formally, we use *G_i_*=<*V_i_*, *E_i_*>, where *i* =1, 2, 3,…, *n*, to represent the *n* networks obtained from corresponding PPI databases where *G_i_* represents a network, *V_i_* represents the set of vertices in a network, and *E_i_* represents the set of edges in a network.

### Traditional union approach

A straightforward approach simply merges the networks using a union operator. More specifically, an integrated network *Ĝ* is obtained as follows:(1)

### Novel *k*-votes approach

Different integration results can be achieved based on how the committee of *n* networks casts votes to decide if an edge should be included into the integrated network. An edge and its associated nodes will be included in the integrated network if and only if a consensus of at least *k* votes is reached in the committee, where *k* can be any number between 1 to *n*. A mathematical representation of this *k*-votes approach is as follows:(2)

where {*G_i_*_1_, *G_i_*_2_, *G_i_*_3_, …, *G_ik_*} consists of a subset of {*G*_1_, *G*_2_, *G*_3_, …, *G_n_*}. As an example, a consensus of one vote (*k*=1) yields an integrated network, which is simply the union of all *n* networks and can be formally represented as follows:(3)

Therefore, the traditional union approach is a special case of our novel *k*-votes approach. Furthermore, a consensus of two votes (*k*=2) returns an integrated network, which can be formally represented as follows:(4)

where {*i*, *j*} is a subset of {1, 2, 3, …, *n*}.

The size of the integrated network shrinks as *k* grows according to set theory [13]. To determine an optimal value for *k*, we used network clustering, or functional module analysis.

### Network clustering algorithm - SCAN

We applied SCAN for functional module analysis [13]. SCAN identifies clusters or functional modules based on structural similarity of a pair of vertices that are connected by an edge. Structural similarity is calculated by using common neighbors. The algorithm tries to assign a vertex to a cluster where it shares many common neighbors with other members of the cluster. More specifically, a vertex is added into a cluster if its structural similarity with a member is larger than a threshold value ε.

### Statistical clustering quality measures

In order to achieve an optimal clustering result, the threshold ε needs to be determined. For this purpose, different ε values such as 0.1, 0.2, … , 0.9 is used for SCAN. This gives a clustering result for each ε value. The quality of the clustering result is then measured by two statistical clustering quality measures, modularity and similarity-based modularity [14].

### Modularity

Modularity is a quality measure of network clustering [15]. Formally, it is defined as follows:(5)

where *NC* is the number of clusters, *L* is the number of edges in the network, *l_s_* is the number of edges between vertices within cluster *s*, and *d_s_* is the sum of the degrees of the vertices in cluster *s*. The value of the modularity measure *Q_N_* ranges from 0 to 1. The optimal clustering is achieved by maximizing *Q_N_*. Modularity is defined such that *Q_N_* is 0 at either extreme of all vertices clustered into a single cluster, or of all vertices randomly clustered.

### Similarity-based modularity

Modularity as a quality measure of clustering leads to resolution limit problem when the size of the clusters varies strongly in networks [16]. More specifically, small clusters are merged by the maximization of modularity, and thus fail to be identified in networks [16]. Feng *et al*. proposed a similarity-based modularity as a remedy, which is defined as follows:(6)

where *NC* is the number of clusters, *IS_i_* is the sum of structural similarity between any connected pair of vertices within cluster *i*, *DS_i_* is the total structural similarity for all vertices in cluster *i*, and *TS* is the sum of structural similarities for all pairs of vertices in the network [14]. Maximization of *Q_S_* yields an optimal network clustering even when the size of the clusters varies strongly. Like regular modularity, the value of similarity-based modularity is between 0 and 1.

### Significance tests

In addition to the two statistical clustering quality measures above, we also perform significance tests to evaluate the clustering results based on the biological meaning. These tests include clustering score and enrichment test, described below.

### Clustering score

The quality of a functional module or a cluster can be measured by the probability *p* that a set of genes are enriched for a given annotation in KEGG by random chance. The *p*-value of an annotation *A* for a cluster measures the chance of seeing that particular cluster or better given the background distribution. More specifically, consider a cluster with size *n*, with *m* proteins sharing a particular annotation *A* and there are *N* proteins in the network, from which *M* genes share the annotation *A*. Then the probability of observing *m* or more proteins that are annotated with *A* out *n* proteins in the cluster is [17]:(7)

The equation above gives the *p*-value of the cluster of proteins for the annotation *A*. The *p*-value is calculated for each annotation in the cluster. A cluster with a minimum *p*-value less than the α level of 0.05 is considered to be a significant cluster. The significance of a clustering result is measured by a clustering score, which is calculated as follows:(8)

where *n_S_* and *n_I_* denote the number of significant and insignificant clusters, respectively [18]. The clustering score is between 0 and 1. The maximal clustering score indicates an optimal clustering outcome.

### Enrichment

We measure the enrichment of KEGG pathway by using cosine similarity between two proteins over the KEGG knowledge base. From KEGG, we extract the annotation vector for each protein. Cosine similarity between two genes *i*, *j* is defined as follows:(9)

where *A_i_* is annotation vector of gene *i*. Enrichment for a clustering result is calculated by using similarity between genes as follows:(10)

where *C_s_* is a cluster of size |*C_s_*|. Enrichment is the average annotation similarity between all pairs of protein that share a cluster divided by the average annotation similarity between all pairs of genes in the network [2]. This enrichment quantifies the quality of all clusters given the domain knowledge from KEGG. To compare enrichment with other quality measures in the same scale we normalize enrichment, so that the normalized enrichment value ranges from 0 to 1.

### KEGG pathway

There are a total of 199 unique human pathways in the KEGG, which involve 5197 unique genes/proteins; the pathway data can be downloaded from http://www.genome.jp/kegg/pathway.html. In this study, the KEGG pathway analysis was performed to investigate whether the biological meanings of modules are significant.

## Results

**Figure 1:**
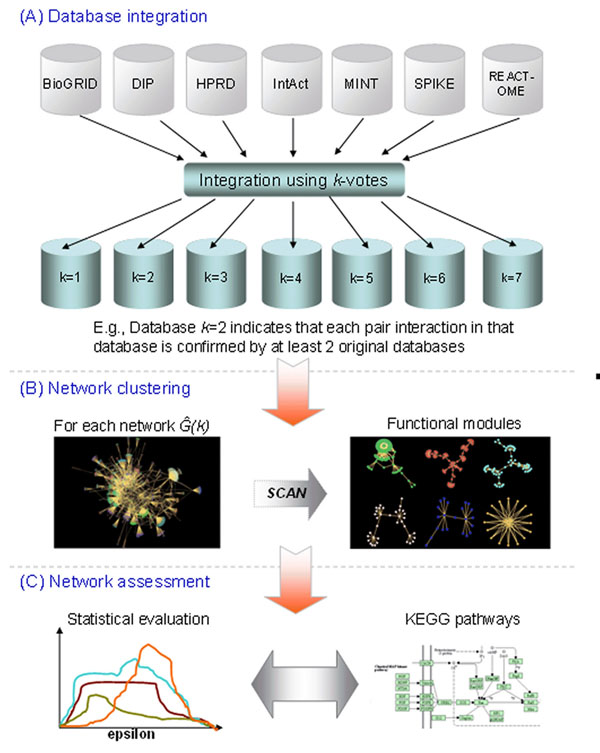
**Network modeling and evaluation flowchart** PPI data are taken from seven preprocessed public PPI databases and used to create seven integrated networks using the *k*-vote method (A). SCAN is used to generate functional modules for each of these integrated networks. (B). Statistical and pathway analyses are performed on these functional modules to assess the networks (C).

The procedure used to integrate multiple PPI databases to yield a modular and biologically meaningful network is shown in Figure 1. Seven PPI databases were preprocessed so that only human data were selected by using unified EntrezGeneIDs. Seven integrated networks were obtained by using the *k*-votes method for *k* = 1, 2, 3, …, *n*, where *n* = 7. In the *k*-votes method, all known interactions are examined, and if an interaction is present in at least *k* PPI databases, it is included in the integrated network.

**Figure 2:**
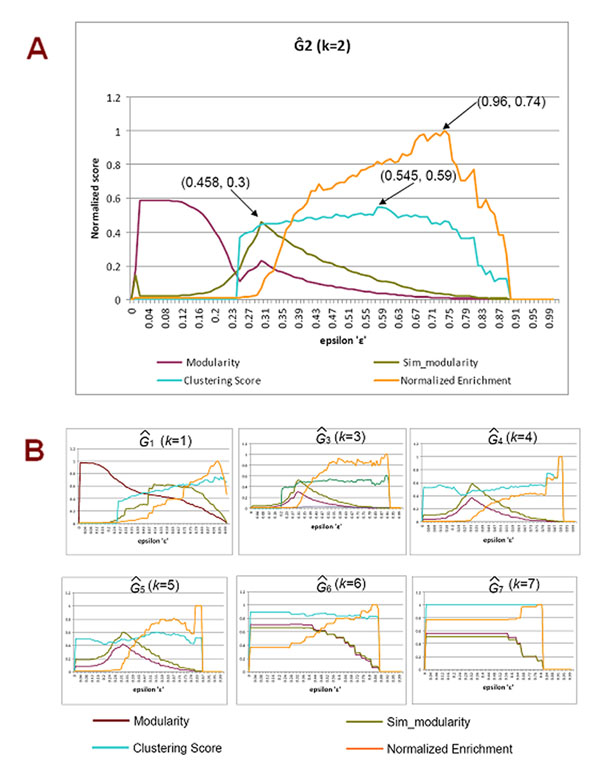
**Optimality measures for the seven consensus networks** Figure 2A shows the four optimality measures for Ĝ_2_: modularity, similarity-based modularity, clustering score, and enrichment score. Figure 2B shows the same measures for the other 6 consensus networks. The value of optimality measures and the corresponding ε values are plotted on the y-axes and x-axes, respectively.

After all seven integrated networks were constructed; cluster analysis was performed on each one using the SCAN algorithm with ε values in steps of 0.01 from 0 to 1. Each ε value yielded a clustering result. We calculated the four quality measures including modularity, similarity-based modularity, clustering score, and normalized enrichment for each clustering result, shown in Figures 2A and 2B. The integrated network that achieved the best overall performance in terms of overall clustering quality measures was recognized as the most informative network.

### Seven integrated PPI networks yielded by using the *k*-votes method

**Ĝ_1_ (*k*=1)**: The network is constructed by including all interactions of seven PPI databases. It is equivalent to the traditional union approach of creating a PPI network. The modularity values show a downtrend over ε and do not reach an optimal value at any ε (Figure 2B). An optimal value for any of the four quality measures is a non-edge case maximal ε value, ε values close to 0 or 1 are not considered because they yield only trivial modules that consist of either all vertices or very few vertices. Similarity-based modularity possesses an optimal value at ε=0.5, which demonstrates a superior performance over modularity. In regards to biological significance tests, both clustering score and normalized enrichment show an uptrend over ε and do not converge to an optimal value. Therefore, we can conclude that network Ĝ_1_ (*k*=1) does not constitute a robust network with a reasonable biological significance. One reason for such results could be due to false positives. Since this network has every interaction proposed by any one of the seven databases, any interaction wrongly identified by even one of the databases would be a false positive and decay the network’s robustness.

**Ĝ_2_ (*k*=2)**: The network comprises interactions that are present in at least two PPI databases. We observed that modularity could not be optimized for any ε value, as was the case for the case of Ĝ_1_ (*k*=1) (Figure 2A). We obtained an optimal similarity-based modularity at ε=0.3, which again demonstrates a superior performance over modularity. In contrast to Ĝ_1_ (*k*=1), there is a clear maximum for both the clustering score and normalized enrichment value, which was at ε=0.59 and at ε=0.74, respectively. Therefore, the network Ĝ_2_ (*k*=2) is both statistically significant and biologically meaningful.

**Ĝ_3_, Ĝ_4_, and Ĝ_5_ (*k*=3, 4, 5)**: For the three networks constructed by using *k*=3, 4, and 5 respectively, we observed an optimality in terms of statistical clustering quality measures including both modularity and similarity-based modularity (Figure 2B). However, there is no biological optimality in terms of either clustering score or enrichment. Therefore, the networks are statistically significant, but not biologically meaningful. Interestingly, we found both modularity and similarity-based modularity were optimized at the same ε value. Since these networks do not possess biological significance, we rule out them as comprehensive networks. One factor that could contribute to the poor biological significance of these networks is the low coverage of interactions, which is the result of high number of votes (*k*) required for the consensus.

**Ĝ_6_ and Ĝ_7_ (*k*=6, 7)**: For networks constructed by using *k*=6 and 7, respectively, the significance tests show flat results over every ε value, which indicates there is neither statistical nor biological significance for both networks (Figure 2B). The main reason behind this is the sparse interactions among proteins; most of the proteins and their interactions are not present in these networks.

	The number of nodes (proteins) and edges (interactions), as well as the presence of optimality, in terms of all quality measures are summarized in Table 2. Based on the results, we concluded that network Ĝ_2_, established by using *k*=2 in the *k*-votes method, is the only one of the seven networks that is both statistically significant and biologically meaningful. Therefore, the best integration strategy is the one using a consensus of at least two votes in the committee of seven PPI databases for this study. On the other hand, the number of edges (interactions) drops by approximately 73% from 132,603 to 36,086, in comparison with Ĝ_1_. Therefore, Ĝ_1_ may be preferred if the coverage of possible protein-protein interactions is more important for the biological study and one is not overly concerned with false positive associations. The significant decrease of interaction coverage also indicates the rarity of agreement between the original seven PPI databases in terms of protein-protein interactions. Hence, there is a trade-off between the coverage and the reliability of protein-protein interactions. The optimal integrated network is a balance that is dependent on the focus of the study.

**Table 2:** Presences of Optimal Quality Measures

	#Nodes	#Edges	Presence of Optimal Modularity	Presence of Optimal Similarity-based Modularity	Presence of Optimal Clustering Score	Presence of Optimal Enrichment
***Ĝ_1_***	12043	132603	No	**Yes**	No	No

***Ĝ_2_***	9188	36086	No	**Yes**	**Yes**	**Yes**

***Ĝ_3_***	6464	17222	**Yes**	**Yes**	No	No

***Ĝ_4_***	4209	8108	**Yes**	**Yes**	No	No

***Ĝ_5_***	2286	3619	**Yes**	**Yes**	No	No

***Ĝ_6_***	345	302	No	No	No	No

***Ĝ_7_***	59	40	No	No	No	No

### Pathway analysis

From a biological perspective, functional modules with high statistical significance reflect a biological (disease) phenotype. The optimal parameter ε=0.59 from the network constructed using *k*=2 achieving the maximal clustering score was applied. 97 out of 158 modules were found to be statistically significant by SCAN using an α level of 0.05. Table 3 lists the top ten modules with significant biological enrichment of KEGG pathways by the clustering score. Proteins with similar biological functions can be successfully clustered together by applying SCAN to the network constructed using *k*=2; in fact, six out of the top ten modules (1, 2, 4, 5, 6, and 8) have a perfect purity for the KEGG pathway represented.

**Table 3:** Top ten modules with significant biological enrichment in KEGG

Cluster ID	KEGG Pathway	Total number of proteins in the module	Number of proteins in the KEGG Pathway from the module	Total number of proteins in the KEGG Pathway	Fisher’s p-value
1	RNA polymerase / Transcription / Genetic Information Processing	10	10	29	5.10E-24
2	Progesterone-mediated oocyte maturation / Endocrine System / Cellular Processes	12	12	86	1.91E-22
3	Proteasome / Folding_ Sorting and Degradation / Genetic Information Processing	17	12	48	5.22E-22
4	Basal transcription factors / Transcription / Genetic Information Processing	9	9	36	1.24E-20
5	Cell cycle / Cell Growth and Death / Cellular Processes	12	12	128	2.97E-20
6	Ubiquitin mediated proteolysis / Folding_ Sorting and Degradation / Genetic Information Processing	12	12	138	7.61E-20
7	Cell cycle / Cell Growth and Death / Cellular Processes	13	12	128	3.78E-19
8	Pyrimidine metabolism / Nucleotide Metabolism / Metabolism	10	10	98	3.57E-18
9	Oocyte meiosis / Cell Growth and Death / Cellular Processes	12	11	114	4.09E-18
10	RNA degradation / Folding_ Sorting and Degradation / Genetic Information Processing	11	9	59	8.97E-17

*the highest level to the lowest level of KEGG pathways (maptitle / subcategory / category) are show

## Discussion

PPI networks play a critical role in many biological studies. While there are many publicly available PPI databases, each source provides a special focus on one type of interaction, and no single source provides a comprehensive view of all interactions. Thus, integration of multiple sources is a promising approach to establish a comprehensive PPI network. In this study, a collection of seven interaction databases is explored for the construction of a robust and biologically significant PPI network. The main contributions are two fold: first, we devised a novel approach, namely *k*-votes, for the integration of multiple interaction networks that were extracted from publicly available sources; second, we developed a network clustering-based framework to determine the best integration strategy, which is defined by the value of *k*.

Recently, Cerami *et al* applied the union approach for the fusion of publicly available pathway data from multiple sources [3]. While the union approach is easy to implement and has maximal coverage of potential interactions, the interactions may not be accurate in the integrated network due to quality issues such as processing errors or missing values in the individual databases. Therefore, the resulting network is not as reliable as our *k*-votes approach using an optimal *k*, where each individual network can be seen as an expert, who has both strengths and weaknesses in terms of the interaction data. Thus, a more robust integration can be achieved based on a partial consensus of the committee of all experts, which consists of individual input databases.

	To determine an optimal *k*, we used several quality measures and performed cluster analysis on the integrated network. The rationale is that a high quality network yields high quality functional modules, which can be determined by quality measures including modularity, similarity-based modularity, clustering score, and enrichment. Therefore, the optimal *k* is estimated by calculating the clustering quality measures for all possible value of *k*. The optimal *k* yields a network that achieves an overall maximum of clustering quality measures. Note that using a higher *k* decreases the number of interactions found in the networks; the increased robustness is achieved at a possible loss of information.

We used the SCAN algorithm for the cluster analysis. Both theoretical and empirical studies show that SCAN can quickly and successfully identify clusters as well as vertices that play special roles (e.g., outliers and hubs) in large networks [5]. In another study, Mete *et al*. applied SCAN for the identification of functional modules in PPI networks [19]. The experimental results demonstrate a superior performance compared to other state-of-the-art algorithms, such as modularity-based algorithms [15].

The modules enriched in the PPI networks were mined to discover new biomarkers related to specific diseases such as breast cancer, diabetes, etc. [20,21]. In this study, our SCAN results yield not only a statistically significant integrated PPI network, but also produce biologically meaningful modules, which are similar to network analysis results from GeneGo (http://www.genego.com/) and IPA (http://www.ingenuity.com/). The enrichment results in Table 3 demonstrate that similar functional PPI can be clustered together.

In summary, this study demonstrates that the integration strategy of using the consensus of two out of the seven databases delivered the best results in terms of both robustness and significance. On the other hand, there is a trade-off between the coverage and the reliability of protein-protein interactions. The maximal coverage can be achieved by using traditional union approach for the integration, which is also a special case of our *k*-votes method (*k*=1). The integration of multiple databases is a promising bioinformatics strategy that can advance knowledge discovery using various public biological databases.

## Conclusions

We determined that the k-votes method for constructing a robust PPI network by integrating multiple public databases outperforms previously reported approaches. Furthermore, our systematic analysis reveals that using a consensus of *k*=2 yields the optimal integration for the seven PPI databases used in this study. The *k*-votes approach holds the potential to improve the integration of other types of networks, such as human disease networks.

## List of abbreviations used

PPI: Protein-protein interaction; SCAN: structural clustering algorithm network.

## Competing interests

The authors declare that they have no competing interests.

## Authors' contributions

MV and ZL performed all calculations, data analysis, and wrote the first draft of manuscript. WT and XX developed the methods, conceived the original idea, and guided the data analysis. LG, ZS, and YY contributed to the construction of the PPI databases and networks. HF contributed to the data analysis and verified the calculations. DD assisted with writing the manuscript. All authors read and approved the final manuscript.

## Disclaimer

The views presented in this article do not necessarily reflect those of the US Food and Drug Administration.
